# Revealing Sources and Distribution Changes of Dissolved Organic Matter (DOM) in Pore Water of Sediment from the Yangtze Estuary

**DOI:** 10.1371/journal.pone.0076633

**Published:** 2013-10-14

**Authors:** Ying Wang, Di Zhang, Zhenyao Shen, Chenghong Feng, Jing Chen

**Affiliations:** 1 The Key Laboratory of Water and Sediment Sciences, Ministry of Education, School of Environment, Beijing Normal University, Beijing, P.R. China; 2 State Key Laboratory of Water Environment Simulation, School of Environment, Beijing Normal University, Beijing, P.R. China; US Naval Reseach Laboratory, United States of America

## Abstract

Dissolved organic matter (DOM) in sediment pore waters from Yangtze estuary of China based on abundance, UV absorbance, molecular weight distribution and fluorescence were investigated using a combination of various parameters of DOM as well as 3D fluorescence excitation emission matrix spectra (F-EEMS) with the parallel factor and principal component analysis (PARAFAC-PCA). The results indicated that DOM in pore water of Yangtze estuary was very variable which mainly composed of low aromaticity and molecular weight materials. Three humic-like substances (C1, C2, C4) and one protein-like substance (C3) were identified by PARAFAC model. C1, C2 and C4 exhibited same trends and were very similar. The separation of samples on both axes of the PCA showed the difference in DOM properties. C1, C2 and C4 concurrently showed higher positive factor 1 loadings, while C3 showed highly positive factor 2 loadings. The PCA analysis showed a combination contribution of microbial DOM signal and terrestrial DOM signal in the Yangtze estuary. Higher and more variable DOM abundance, aromaticity and molecular weight of surface sediment pore water DOM can be found in the southern nearshore than the other regions primarily due to the influence of frequent and intensive human activities and tributaries inflow in this area. The DOM abundance, aromaticity, molecular weight and fluorescence intensity in core of different depth were relative constant and increased gradually with depth. DOM in core was mainly composed of humic-like material, which was due to higher release of the sedimentary organic material into the porewater during early diagenesis.

## Introduction

Aquatic sediments are considered invaluable natural archives that provide long-term records of past changes in environment and also register anthropogenic activities and man-made environmental problems [1], [2]. When the environmental conditions change, adsorbed matters on the sediments could be desorbed into pore water and then diffuse into overlying water, which would lead to the change of aquatic environment [3]. So pore water as a medium connecting sediments and overlying water is important for the identification of biogeochemical processes in aquatic systems.

Dissolved organic matter (DOM) is an important component in the natural aquatic systems [4], especially in marine and coastal environments. Substantial terrestrial DOM inputs through river discharges are estimated to contribute 0.25×10^15^ g carbon (C) yr^−1^ to the ocean carbon pool [5]. Together with the terrestrial DOM, rivers convey nutrients, which can result in intense primary productivity, leading to the production of autochthonous DOM [6]. As an important marine system, estuarine ecosystem has always been hot spot of DOM cycling because its DOM composition is controlled by the relative abundance of many different DOM sources, allowing the transfer of organic or inorganic substances from the continental to the oceanic environment. So DOM in the estuary has significant effect in substance cycling because of its high primary productions as well as the complex interactions of physical, photochemical and microbial processes.

As one of the world’s largest estuaries, the Yangtze estuary situated in east China, containing a dense population of 13 million and concentrated industry [7]. The Yangtze estuary is the super subtropical muddy estuary with high spatial heterogeneity and complex DOM sources [8]. It was reported that the Yangtze River carries fine sediments 480 Mt yr^−1^ to the sea [9]. Wherein, over half of these materials are deposited in the Yangtze estuary. In recent years, flow rate and river sediment discharge downstream the Yangtze River has dramatically decreased because of the construction of dams and large waterway projects in the Yangtze River [10], resulting in the changes of material cycle processes in the estuary. So the study on the chemical characteristics and variations of DOM in the Yangtze estuary has extensive implications in aquatic ecology.

There has been a great quantity of researches in the distribution and characterization of DOM in pore water from estuarine and coastal systems over the past decades [2], [4], [11]–[13]. To date, only few studies have, to the best of our knowledge, been carried out on the characterization of pore water DOM comprehensively in the Yangtze estuary. In their studies, only traditional DOM coefficients and ‘peak picking’ technique of EEMs were used, and thus DOM variations couldn’t be clearly indentified [8], [14]. Nowadays, the environmental dynamics (i.e., source and fate) of DOM components in aquatic ecosystems have been evaluated by using an advanced approach, the combined techniques of excitation–emission matrix (EEM) fluorescence with parallel factor analysis (PARAFAC) [6], [15]. The EEM-PARAFAC can be a powerful method for detecting small but potentially significant variations in DOM composition quickly and conveniently. It was considered an ideal technique for understanding DOM dynamics in water systems [16], [17].

Therefore, our present study concerns quantitative and qualitative characterization of pore water DOM in the Yangtze estuary of China by using EEM-PARAFAC methods. The objective of this study was to (1) characterize the vertical and horizontal spatial distributions of the components and abundance of sediment pore water DOM, to realize its biogeochemical nature and properties. (2) explore the origin of pore water DOM, further to understand the contributions of internal release or anthropogenic activities to the fluorescent organic matter in sediment interfaces.

## Materials and Methods

### Study Area and Sample Collection

The Yangtze estuary is the largest estuary in China [18]. In the Yangtze estuary, the Yangtze River is divided into two large river systems (southern branch and northern branch) by Chongming island based on the island distribution characteristics [9]. The South Branch constitutes the main stream of the estuary and receives above 95% of the total estuarine runoff, whereas the North Branch accounts for only about 5% [19]. Therefore, the South Branch was the mainstream of river channel in the Yangtze estuary.

**Figure 1:**
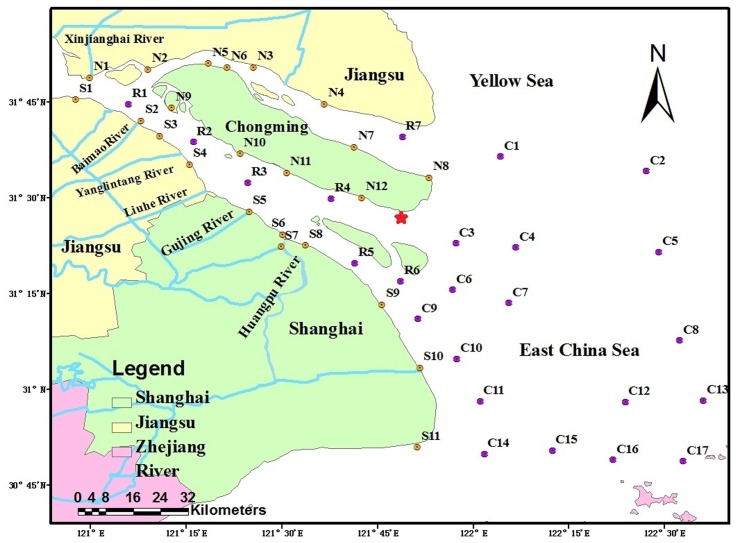
Map of sampling sites from the Yangtze estuary. The southern nearshore samples (S1–S11), northern nearshore samples (N1–N12), river channel samples (R1–R7) and coastal areas samples (C1–C17).

According to the spatial distribution (Fig. 1), a total of 47 sites in four regions (northern nearshore, southern nearshore, river channel, coastal area) were chosen to collect surface sediment in May and August 2010. Wherein, 11 sites along the southern branch nearshore (S1–S11), 12 sites along northern branch nearshore and around Chongming Island (N1–N12), 7 sites from south branch (R1–R7) of river channel and 17 sites from the coastal areas (C1–C17). The surface sediments were sampled to a depth of 0–2 cm using a Van Veen stainless steel grab sampler (Eijkelamp, Netherlands).

The sediment core was obtained at the outlet of the south branch (the asterisk in Fig. 1) using a gravity corer (with a tube of 10 cm in diameter and 30 cm in length). The core was undisturbed during the sampling processes and then sliced into 2 cm sections under N_2_ atmosphere. 13 samples were obtained from sediment core. All 60 Samples were then centrifugated to extract pore waters at 5000 rpm for 10 min. All water samples were filtered through 0.22 µm pre-combusted fiberglass filters and then stored in amber glass vials in the dark at 4°C until they were analyzed.

### Characterization of DOM in Pore Water

#### Optical measurements

The UV absorption spectra were analyzed on a Cary 50 UV–Visible spectrophotometer (UV-VIS) (Varian, Inc., Australia) by using Milli-Q water to make base line, scanning 700∼200 nm by 1 nm in medium rate. Samples were put into 1 cm quartz sample cell after stabilized. A blank scan (Milli-Q) was subtracted from each spectrum. The blank corrected sample spectra were baseline corrected by subtracting values of 700 nm from the entire spectrum, and then converted to spectral absorption coefficients, a (λ, m^−1^) [20].

Three-dimensional excitation–emission matrix (EEM) fluorescence spectra were scanned by a Hitachi F-4600 fluorescence spectrometer (Hitachi High-Technologies, Tokyo, Japan). The equipment parameters are set as PMT voltage = 700 V; bandpass: Ex = 5 nm, Em = 5 nm; respond time = 0.5 s; scan rate = 2400 nm/min; Ex is from 200 nm to 400 nm by 5 nm, while Em is from 290 nm to 550 nm by 3 nm. Samples were placed in a 1 cm path length, fused silica cell and blank sample is Milli-Q water. The water Raman peaks and Rayleigh scatter effects were removed according the method reported by Yao [21].

#### Optical indices

The UV absorbance is usually related to DOM. Because of the chemical complexity of DOM, the adsorption coefficient at 355 nm (a_355_ m^−1^) was reported as a quantitative parameter for DOM abundance [22].

One simple indicator, SUVA, was used to characterize and differentiate DOM. It was determined by dividing the absorbance a_254_ by the corresponding DOC concentration. Relatively higher SUVA values (>3 Lmg^−1^m^−1^) are associated with higher degree of aromaticity and unsaturation [23].

According to some previous studies, the ratio of the fluorescence emission intensity at 450 nm to that at 500 nm with Ex = 370 nm is an index which distinguishes the origins of DOM [17]. Higher FI values always mean higher autochthonous to allochthonous ratios or higher proportions of the aromaticity-poor fulvic substances.

#### DOC measurements

DOC concentrations of all pre-filtered pore water samples were determined by a TOC-VCPN organic carbon analyzer (Shimadzu, Co., Japan) using combusting oxidation with a platinum catalyst in 800°C. The apparatus was calibrated using a standard solution of HOOC–C_6_H_4_–COOK.

#### Molecular weight measurements

The molecular weight distribution was measured by an Agilent PL-GPC 50 integrated Gel Permeation Chromatograph (GPC) system (Agilent Technologies, USA) following the procedures of Fu [4] and Wang [24]. The GPC system was equipped with a pump in isocratic mode with a flow rate of 0.6 ml·min^–1^, pressure of 1.76 MPa and a UV detector set at a wavelength of 254 nm. The mobile phase was composed of 0.002 M Na_2_HPO_4_, 0.002 M NaH_2_PO_4_ and 0.1 M NaCl. The calibration of the molecular weight was based on polystyrene sulfonate (PSS) standards (13.65, 6.2, 4.6, 3.42 KDa), and Acetone (58 Da); (American Polymer Standards, Mentor, OH). The number-averaged molecular weight (Mn) and weight-averaged molecular weight (Mw) for the porewater samples were calculated using the following equations [4]:
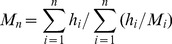
(1)

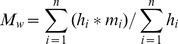
(2)where *M_i_* is the molecular weight at eluted volume *i*, and *h_i_* is the height of the sample HPSEC curve eluted at volume *i*.

### The PARAFAC Modeling

PARAFAC was used to model the dataset of F-EEMs. It statistically decomposes the complex mixture of DOM fluorophores into components about their spectral shapes or numbers, thus allowing the estimation of the true underlying EEM spectra. The approach of PARAFAC modeling to EEMs has been described in detail elsewhere [25], [26] and is briefly described here. PARAFAC decomposes N-way arrays into N loading matrices. That is, if fluorescence EEMs are arranged in a three-way array *X* of dimensions *I*×*J*×K, where *I* is the number of samples, *J* the number of emission wavelengths, and *K* the number of excitation wavelengths, PARAFAC decomposes them into three matrices A (the score matrix), B and C (loading matrices) with elements *a_if_*, *b_jf_*, and *c_kf_* [15].

### Statistical Analyses

In this study, PARAFAC analysis was carried out using MATLAB 7.0 (Mathworks, Natick, MA) with the DOMFluor toolbox [16]. The data set was composed of 47 samples from the four regions of surface sediment pore waters and 13 core samples. A series of PARAFAC models consisting of between three and seven components were computed for the EEMs [25]. The final correct number of components was primarily achieved by several methods of split half analysis, residual analysis, and visual inspection [25].

SPSS 13.0 software was used for statistical analyses (mean value, standard deviations), correlation analyses and principal component analysis (PCA) in this study. Significance levels are reported as non-significant (p>0.05), significant (0.05>p>0.01) or highly significant (p<0.01).

## Results and Discussion

### Distribution and Variations in Quality of DOM in Pore Water

#### Horizontal distribution of DOM quality in four regions of the Yangtze Estuary

The properties of DOM in pore water samples collected from four regions of the Yangtze estuary were analysed (Table 1). In the Yangtze estuary, the values of DOC and a_355_ were obviously variable, ranging from 20.3 to 448.3 mg/L and 34.7 to 289.4 m^−1^, respectively. The mean values of DOC and a_355_ were higher and more variable in the nearshore areas than that in the other two areas, especially in south nearshore. This result may be due to the input of anthropogenic pollutants from the nearby regions [2]. Additionally, there was a significant correlation (*r* = 0.895, *p*<0.01) between DOC and a_355_, which was parallel to previous studies [27], [28]. This result indicated that DOM in pore water occupied the main part of DOC in Yangtze Estuary [28]. Higher values of DOC and a_355_ in pore water were observed at the sites S5, S7 and S8 in south nearshore area. The sites S5 and S7 are nearby the inflow of tributaries (Gujing river and Huangpu river), where a large number of sewage was input by these tributaries. The site S8 is located near the petrochemical dock, where oil spill directly caused organic pollution. Therefore, higher values in the pore water may be attributed to the increased pollutants input from the tributaries and petroleum discharge.

**Table 1:** Variation of DOM quality indices of samples from four regions of the Yangtze estuary.

		South Branch	North Branch	Riverine	Coastal Area
DOC(mg/L)	max	448.3	334.5	167.0	122.4
	min	51.3	18.3	36.4	20.3
	mean	146.0	91.4	88.0	60.0
a_355_ (m^−1^)	max	289.4	169.7	110.1	152.8
	min	62.5	34.7	38.4	27.5
	mean	151.1	92.3	66.6	63.3
SUVA (L·m^−1^mg^−1^)	max	5.02	2.02	1.28	2.33
	min	0.64	0.38	0.59	0.49
	mean	1.46	1.13	0.95	1.12
Molecular Weight (M_w_) (Da)	max	3771	3771	3729	3729
	min	253	892	847	934
	mean	2952	2546	2546	2191
FI	max	1.93	1.84	1.80	2.24
	min	1.67	1.70	1.65	1.68
	mean	1.75	1.77	1.73	1.84

The SUVA index was used to reflect the humic composition change of DOM. The average SUVA in the Yangtze estuary was 1.19±0.47 L·mg^−1^·m^−1^ (lower than 2 L·mg^−1^·m^−1^), an indication of lower aromaticity [23]. The molecular weight of DOM ranged from 252.6 to 3771 Da, most of which were lower than 3000 Da, indicating that DOM in pore water of Yangtze estuary was mainly composed of low molecular weight materials. The levels of SUVA and molecular weight were higher and more variable in the southern nearshore areas, meaning that samples in the southern nearshore were characteristic of higher molecular weight and degree of humification than that in the other areas. The widely variation of aromaticity and molecular weight in southern nearshore may be primarily due to the influence of frequent and intensive human activities in this area.

The fluorescence index (FI) has been proposed as a proxy to assess the microbial versus higher terrigenous contribution to the fulvic acid pool in DOM [29]. The values of FI in this study ranged from 1.37 to 2.24 and the most generally varied between 1.7 and 1.8 (Table 1), suggesting a combination contribution of microbial DOM signal and terrestrial DOM signal [16]. A similar range of FI was reported for the Yangtze estuary in other reports [14]. The lowest FI value of 1.37 was found in site S5, suggesting heavily terrestrial input. The highest FI value (in site C13) was higher than 2, which was an indication of intense microbial activities.

#### Vertical distribution of DOM quality in sediment core of the Yangtze estuary

**Figure 2:**
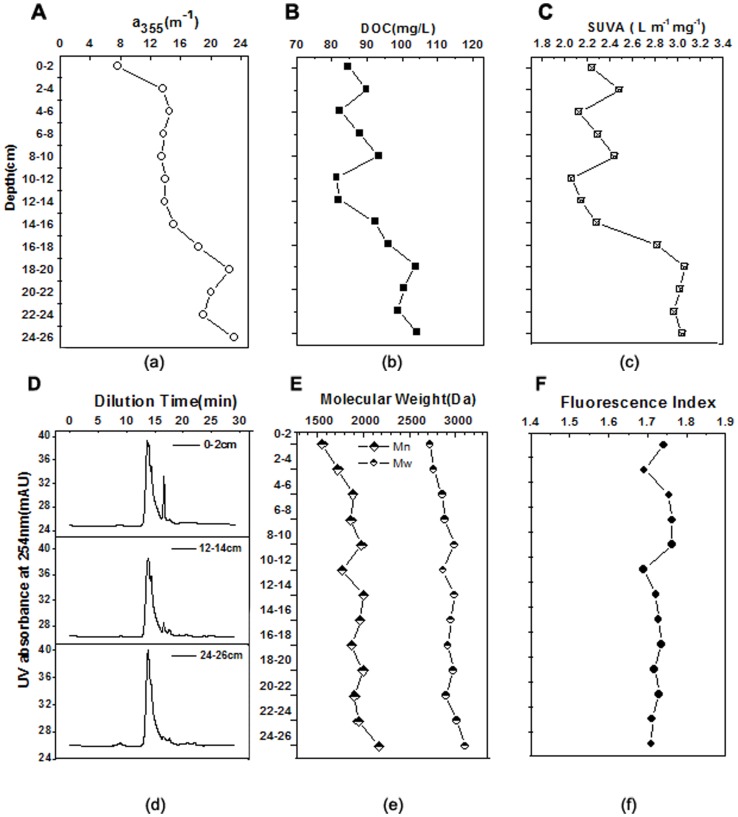
The quality parameters of DOM in core from Yangtze estuary. (A) UV absorption coefficients at 355 nm (A355); (B) DOC concentrations; (C) absorbance at 254 nm/DOC ratios, L m^–1^ mg^–1^(SUVA); (D) size exclusion chromatograms in different sediment depths of core; (E) The number-averaged molecular weight (Mn) and weight-averaged molecular weight (Mw). (F) fluorescence index (FI).

The quality parameters of DOM in sediment core were shown in Fig. 2. The mean value of a_355_, DOC and SUVA were 15.4±3.11 m^−1^, 98.5±8.17 mg/L and 0.58±0.16, respectively, which were less variable than those in the pore water of surface sediment from four regions. The variation of a_355_, DOC and SUVA exhibited the same trend. That is, they increased gradually from the sediment–water interface down to the deeper layer, indicating that DOM contents and humic degree increased with depth. This trend is similar to the pore water quality profile from other reports [30]–[32]. The higher DOM concentration in deep layer can be from the accumulation of primarily humic substances formed by abiotic polymerization of low-molecular-weight DOM or anaerobic degradation of particulate organic matter [4], [32].

All porewater DOM samples from core exhibited multimodal HPSEC chromatograms with sharp and well resolved peaks (Fig. 2D). The chromatograms were similar to those of porewater DOM from sediments of the northern basin of Lake Michigan [33] and core water in Lake Võrtsjärv [2]. The majority of the core water DOM from Yangtze estuary was of relatively low molecular weight (<3000 Da). Mw ranged from 2724 to 3104 Da, and Mn from 1558 Da to 2170 Da. There seemed to be a slight increase in Mw and Mn with depth (Fig. 2E). The ranges and trends of core in Yangtze estuary agreed well with reported for the porewater DOM from Lake Erhai [4] and Lake Võrtsjärv [2]. It was reported DOM in the oxic upside sediments was composed of relatively small organic compounds while DOM in deeper suboxic sediments may contain much larger molecular materials [32].

The values of FI in core ranged from 1.70 to 1.78 (Fig. 2F), an indication of the combination of microbial DOM signal and terrestrial DOM signal [16]. It also suggested that the DOM consisted of compounds which remained relative constant [4].

### EEM–PARAFAC Components of DOM

**Figure 3:**
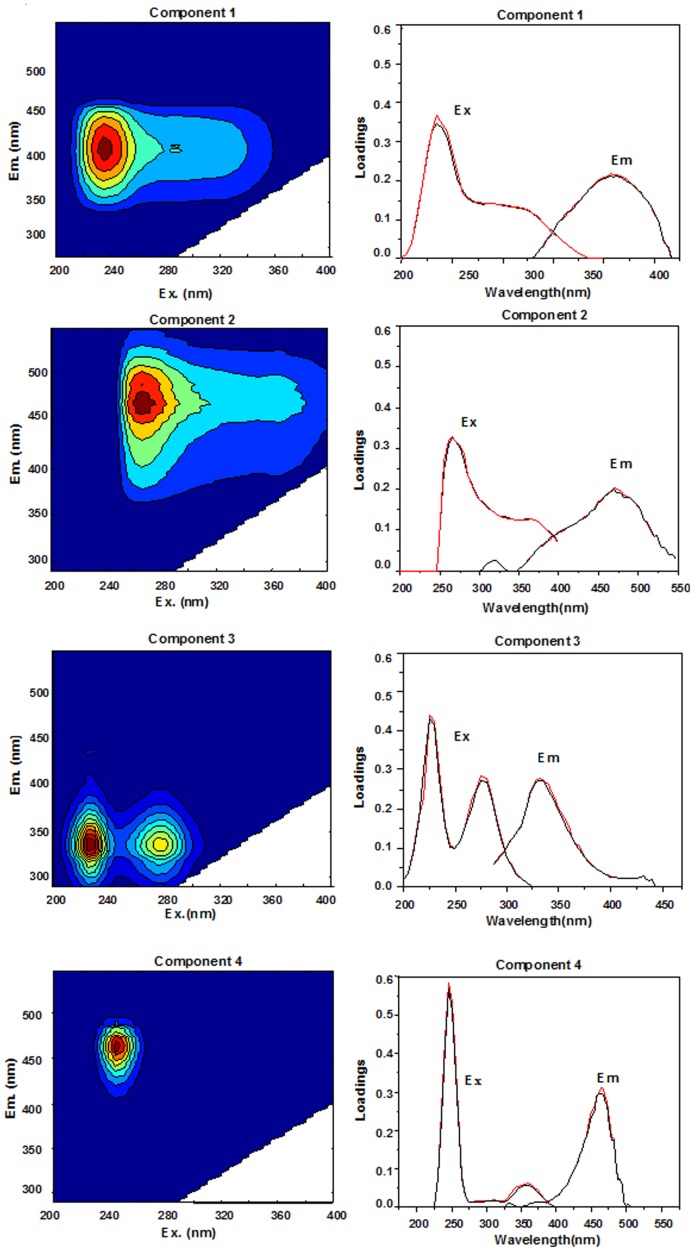
Contour plots of the 4 components identified from the PARAFAC model. The line plots on the right side of contour plots show split-half validation results of excitation and emission loadings between the complete dataset (red) and one of the independent halves (black).

In this study, 60 EEMs of water samples from different regions and core were decomposed into four fluorescent components (C1–C4) by PARAFAC analysis. As shown in Fig. 3, three humic-like substances (Component 1, Component 2, Component 4) and one protein-like substance (Component 3) were identified.

The C1 component was characterized by peaks at 230 nm and 300 nm excitation with emission 410 nm emission wavelengths. This component was categorized as mixture of the traditional terrestrial humic-like peak A and marine humic-like peak M [13], [34], [35]. The spectral features were agreed with the PARAFAC components of terrestrial humic-like materials and terrestrial-marine humic-like materials [6], [36].

In the C2 component, there were two excitation maxima at 265 nm and 365 nm with emission at 344 nm, which was categorized as the mixture of the traditional terrestrial humic-like peaks A and C. The wavelengths agreed with the identified terrestrial-derived humic-like PARAFAC component in other reports [6], [21], [37].

The C3 component was composed of two peaks with excitation maxima at 225 nm and 275 nm with emission at 335 nm which were confirmed as an autochthonous tryptophan-like fluorescence peak T [34], [35]. It agree with a protein-like fluorescent compound in PARAFAC components [36], [37].

The EEM spectral characteristics of C4 was characterized by peaks at 250 nm excitation with 450 nm emission wavelengths, which was similar to a terrestrial humic-like fluorescence peak A [34], [35], [38] traditionally defined in DOM, and the PARAFAC components of terrestrial humic-like materials [6], [36].

### Spatial Distribution of PARAFAC Component Scores

**Figure 4:**
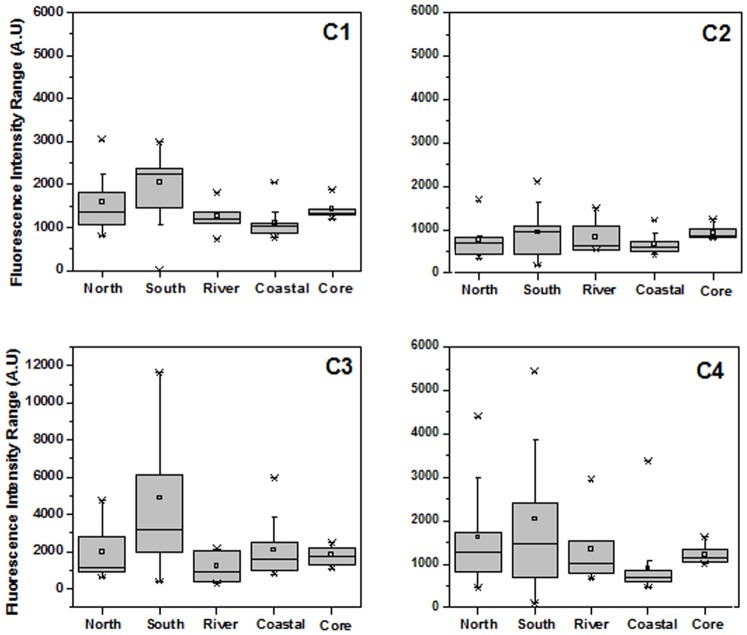
Box plots of DOM fluorescence of four components by PARAFAC (C1–C4) in five regions.

The spatial distribution of each PARAFAC component is shown in Fig. 4. The box plots showed the variability and mean values of fluorescence intensity of pore water in four regions and the core of the Yangtze estuary.

The intensities of four components in the southern nearshore were significantly higher and greater variable than that in other region samples. This result was similar with that of DOM abundance, the degree of humification and molecular weight discussed in the section of 3.1.1. The results suggested that intense anthropogenic activity and tributaries input may contribute to a great deal to the fluorescence intensities of DOM in the Yangtze estuary [8].

In our study, the spatial distribution of C1, C2 and C4 in different regions were similar, with an intensitiy order: southern nearshore > northern nearshore > river > coastal area, indicating that these three components had a common source. This was consistent with the result of the fluorescence characteristics in Fig. 3, showing C1, C2 and C4 were both identified as humic substances components. The behavior of C3 as a tryptophan-like fluorescence peak was different from that of humic-like components, with an intensitiy order: southern nearshore > northern nearshore > coastal area > river. The mean fluorescence level of C3 in coastal area was higher than that in river channel, suggesting that higher autochthonous microbial activity may occurred in coastal area and contribute to a great deal of tryptophan-like fluorescence of DOM [8].

The mean values of fluorescence intensity in the core were lower than that in the nearshore areas and higher than that in the river and coastal samples. Compared to four regions, fluorescence components in the core were less variable. This may be due to DOM in the core were less influenced by anthropogenic activities, leading to more similar structures in vertical depth.

**Figure 5:**
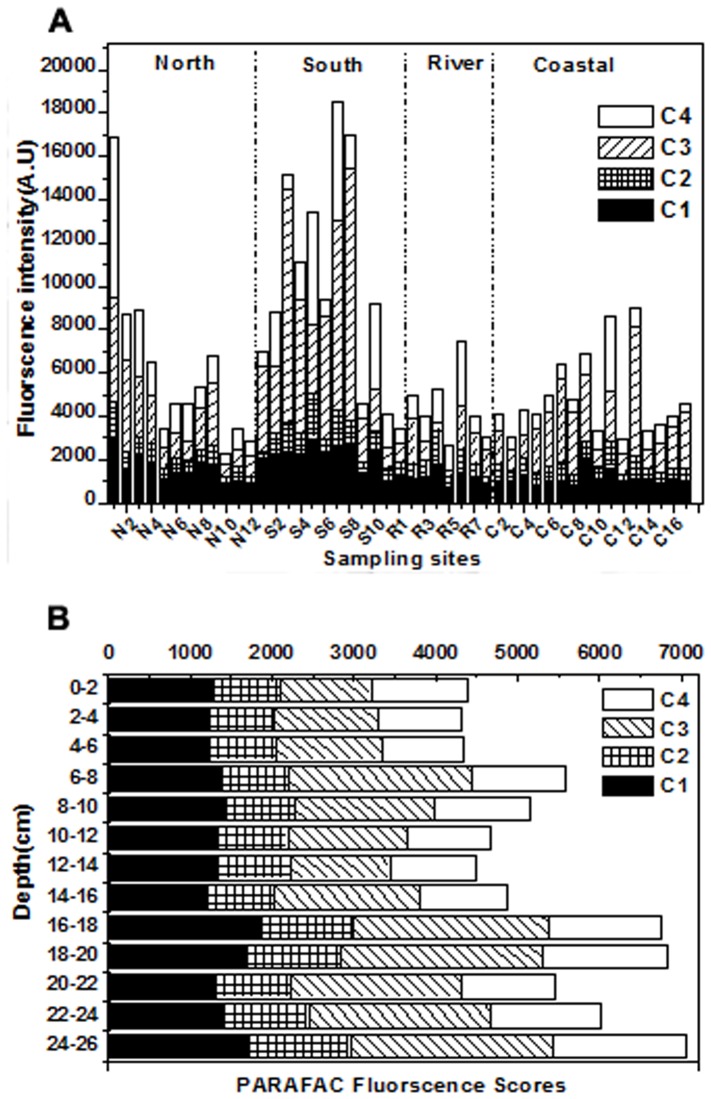
Distributions of DOM PARAFAC scores for all the samples in the Yangtze estuarty. (A) Surface sediment pore water samples of four regions. (B) Core samples in different depth.

Scores of each component for all the pore water samples were shown in Fig. 5. Higher intensities of the protein-like component C3 than humic-like components were observed in the most samples, indicating that DOM in pore water of the Yangtze estuary was dominant by protein-like materials. Total PARAFAC scores in the southern nearshore were the highest and the greatest variable among four regions. Higher values were observed at the sites N1, S3, S7 and S8. Wherein, the highest score was found in site S7, which was nearby the inflow of Huangpu River. N1 was located in the inflow of Xinjianghai River and affected by water incursion from southern branch. Therefore, tributaries inflow was an important factor influence DOM fluorescence distribution. The sites S3 and S8 are nearby the harbor and dock (Xinhe harbor and petrochemical dock), indicating that a large number of organic material was input by shipping.

For DOM in the pore water from the sediment core, there was an increasing trend along 0 to 26 cm depth (Fig. 5B), which was due to higher release of the sedimentary organic material into the pore water during early diagenesis [4]. The fluorescence intensities of each component in the pore water exhibited similar trends as those of the DOC concentration (Fig. 2B) and the UV absorbance (Fig. 2A), which was consistent with the results from other reports [32]. The fluorescence intensities in 6–8 cm and 16–20 cm increased significantly, which may be due to intense microbial activities caused by pollution of diagenesis years [4].

### Principal Component Analysis (PCA) of PARAFAC Component Distributions

**Figure 6:**
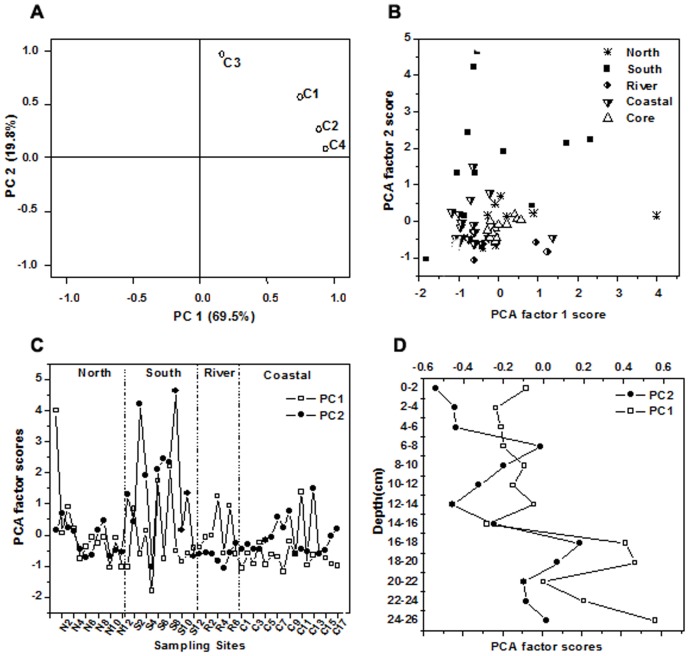
PCA analysis of PARAFAC components. (A) Property–property plots of PCA factor loadings; (B) property– property plots of PCA factor scores of all samples; the changes of PCA scores for (C) surface sediment pore water samples of four regions. (D) core samples in different depth.

To comprehensively assess DOM spatial differences, Principal Component Analysis (PCA) was carried out using the relative a bundance of the four PARAFAC components (Fig. 6). For all samples, the first and second axes of the PCA (factors 1 and 2, respectively) accounted for 69.5% and 19.8% of the variance in PARAFAC component distribution, respectively. The PCA scores were plotted on the first two axes separately for each site to explain the variation tendency of parameters. Each PCA factor is a linear combination of four PARAFAC components:
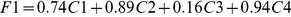
(3)

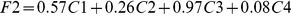
(4)


Fig. 6(A) showed the property–property plots between the first and second factor loadings. All four parameters showed positive factor 1 loadings. Wherein, the three humic-like components (C1, C2 and C4) were close to the factor 1 axis and far from the factor 2 axis. The tryptophan-like component C3 showed highly positive factor 2 loadings but was less significant related to factor 1 axis than the other three parameters. Thus, the PARAFAC–PCA could separate the source contribution characteristics between the humic-like components and the protein-like components in the total fluorescence analyses [28].

The first and second principal component scores of all 60 samples are plotted in Fig. 6(B). The scores of the southern nerashore samples were more scattered, while the samples in the coastal area were more relatively clustered. This result indicated that the composition of DOM was more variable in the southern nearshore than that in other regions. Most of the coastal area samples were clustered with lower factor 1 and higher factor 2 scores, with PCA scores ranging from −1.17 to 1.37 for PCA 1 and −0.61 to 1.47 for PCA 2, while there was no apparent pattern for most of the samplesin other three regions. Obviously, it can be identified that DOM in coastal area was mainly influenced by factor 2.

Samples in the core were much clustered, indicating that DOM structures in core were less variable. PCA scores in the core were more scattered in PCA 1 axis and relative more clustered in PCA 2 axis, with PCA scores ranging from −0.28 to 0.57 for PCA 1 and −0.58 to 0.19 for PCA 2. The lower factor 2 and higher factor 1 scores suggest that samples in the core may be mainly dominated by factor 1.

In order to discuss the source and characteristic changes in each component for all the water samples, Fig. 6(C,D) were conducted. For the most of the southern nearshore samples, PCA factor 2 scores were higher than PCA factor 1 except the site S2 and S11. This result showed DOM in pore water of southern nearshore were mainly influenced by tryptophan-like materials. This was consistent with the result from Fig. 4. Protein-like fluorescence was mainly derived from the degradation of microbial in natural water body. It could also increase if water was polluted [22]. In our study, the PCA factor 2 was higher in the regions affected by intensive anthropogenic activity such as tributaries inlets (S6 and S8), docks (S3,S6 and S8). That is, sewage and runoff from tributaries, anthropogenic activities such as shoreside discharge and shipping were dominant source for DOM in southern nearshore during our sampling dates.

Previous studies have reported that the eutrophic trend was increasesd downstream the Yangtze River after the establishment of the Three Gorges Project [39]. Wherein, the growth, reproduction and decomposition of phytoplankton in eutrophication can influence the abundance and composition of protein. For this reason water conservancy construction such as Three Georges Project (TGP) may also have impact on protein-like distribution in the region. Therefore, due to the large-scale exploitation, human activities and project construction, large contents of protein-like materials have been transported to the sediment of the southern nearshore in the Yangtze estuary, leading to higher protein-like fluorescence in pore water.

Most samples in coastal area clustered with lower first and higher second PCA scores (approximately −1.17 to 1.37, and −0.61 to 1.49, respectively). Owing to little effects of human activities, DOM in pore water of the coastal area may be mainly derived from autochthonous microbial activities [21]. For the most of the northern nearshore samples, few differences were existed between PCA factor one and two scores, suggesting that there are both terrestrial and autochthonous DOM inputs in northern nearshore.

Unlike the other three regions, the PCA factor 1 scores were higher than factor 2 for all the river channel samples except the site R7. This result showed that DOM in river channel were mainly influenced by humic materials, which may be attributed to the mobilization of particulate organic matter (POM) in riverine suspended sediments during the processes of inflow from Yangtze River and sediment resuspension [8].

As shown in Fig. 6(D), The PCA scores of both factor one and two in the core exhibited the same trend in vertical direction. The PCA factor scores increased with the gradual increasing of depth, which was consisted with the profile trend of pore water in previous studies [4], [13]. The PCA results showed a higher relative abundance of humic-like fluorescence components compared to tryptophan-like except site of 6–8 cm, indicating a humic dominating source in core. The PCA factor two in 6–8 cm was higher than factor 1, which may be caused by microbial pollution of diagnesis years. The sampling site of core in our study was near northern site N7 in Chongming island where was less influenced by anthropogenic activities. Therefore, the release of the sedimentary organic material into the pore water during early diagenesis may dominate the DOM in core [4].

## Conclusions

In this study, a combination of various parameters of pore water DOM as well as PARAFAC-PCA analysis clearly identify the degree of variability and separate the source of the DOM in different regions of the Yangtze estuary. DOC in pore water of Yangtze estuary was very variable, ranging from 20.3 to 448.3 mg/L and mainly composed of low aromaticity and molecular weight materials. Three humic-like substances (Component 1, Component 2, Component 4) and one protein-like substance (Component 3) could be identified by PARAFAC analysis. Protein-like component C3 dominant the fluorescence composition of DOM. The PCA analysis of the relative abundance of PARAFAC components showed a combination contribution of microbial DOM signal and terrestrial DOM signal in the Yangtze estuary.

Higher and more variable DOM abundance, aromaticity and molecular weight of surface sediment pore water DOM can be found in souernthern nearshore than other regions, with an content order: southern nearshore > northern nearshore > coastal area > river. DOM in southern nearshore and coastal areas were significantly influenced by tryptophan-like materials. There was a mixing origin in northern nearshore and a humic-like dominating source of DOM in river channel. Due to the large-scale exploitation, human activities and project construction, large contents of protein-like materials have been transported to the sediment of the southern nearshore in the Yangtze estuary, leading to higher protein-like fluorescence pollution in pore water.

The DOM abundance, aromaticity, molecular weight and fluorescence intensity in core of different depth were relative constant compared to DOM in surface sediment pore waters and increased gradually from the sediment–water interface down to the deeper layer. A humic-like source dominating the DOM structures in core, which was due to higher release of the sedimentary organic material into the porewater during early diagenesis.
